# Bone Remineralization around Dental Implants following Conservative Treatment after Peri-Implantitis

**DOI:** 10.1155/2019/7210837

**Published:** 2019-09-05

**Authors:** Algirdas Puisys, Viktorija Auzbikaviciute, Renata Simkunaite-Rizgeliene, Dainius Razukevicius, Rokas Linkevicius, Tomas Linkevicius

**Affiliations:** ^1^Vilnius Implantology Center, Vilnius, Lithuania; ^2^Vilnius Research Group, Vilnius, Lithuania; ^3^Vilnius University, Vilnius, Lithuania; ^4^Lithuanian University of Health Science, Kaunas, Lithuania

## Abstract

The aim of this case report is to show that bone remineralization around dental implants with a history of peri-implantitis is possible after irritant factors are removed and only conservative treatment is performed. Patient came to the clinic after three years of dental implant placement complaining about swelling, sensitivity and gingiva color changes at the posterior part of the maxilla. During radiographic and intraoral examinations peri-implantitis of the #24 implant site was diagnosed. The surgical treatment method was rejected and performed conservative treatment instead. The outcome is promising; periapical radiographs three months later showed bone remineralization as well as stable bone after 10 years. *A key clinical message*: Bone remineralization around dental implants with a history of peri-implantitis is possible after irritant factors are removed and conservative treatment performed.

## 1. Introduction

Biological complications predisposed by undetected cement remnants are receiving much attention recently. It was shown that excess cement might be responsible not only for quick peri-implantitis development but also for delayed or chronic manifestation of the disease many years after cementation [1, 2]. In vitro and clinical studies show that it is very difficult or in some cases even impossible to completely clean up cement excess from subgingival margins—the most popular position of a cementation line—when cement-retained restorations are fabricated [3–5]. The outcome of biological complications due to cement excess may vary from temporary inflammation of soft peri-implant tissues without serious esthetic and functional consequences to implant loss. There is a variety of scientific evidence that described treatment modalities to prevent or suppress the disease. However, there is still the lack of information about nonsurgical treatment and bone remineralization around implants.

This case report describes peri-implantitis caused by residual cement and the solution of the complications and provides a nonsurgical approach of the treatment.

## 2. Case History

**Figure 1:**
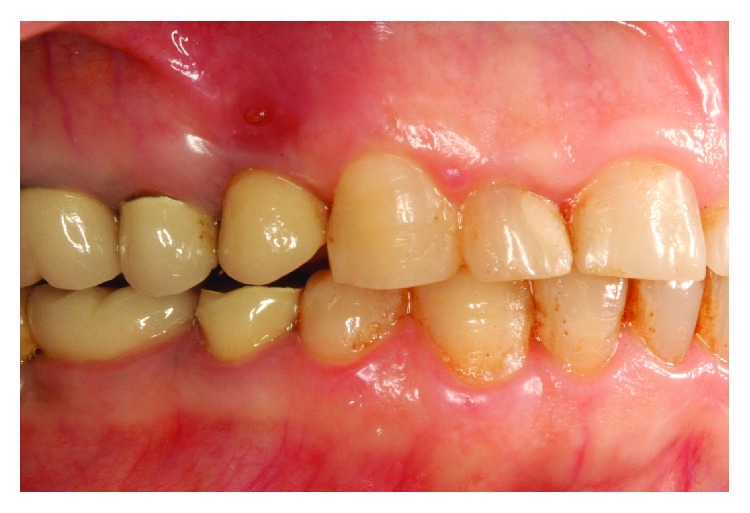
Draining sinus tract over implant-supported restoration.

A patient presented in 2009 with the main complaint of a fistula and tenderness on chewing and touching of the tissues above the implant restoration (Figure 1). Anamnesis revealed that this case was restored approximately 3 years before.

The patient's history chart showed that a regular connection two-piece implant (BioHorizons Internal, Birmingham, AL, USA) was placed and achieved successful integration. A standard abutment was used to support metal ceramic restoration, which was cemented with glass-ionomer modified with a resin luting agent (Fuji Plus, GC, Tokyo, Japan). It was recorded in that cement remnants were cleaned, and radiographic examination did not show any residue. The treatment was considered being finished; the patient was satisfied and did not seek any consultation or intervention after the treatment.

**Figure 2:**
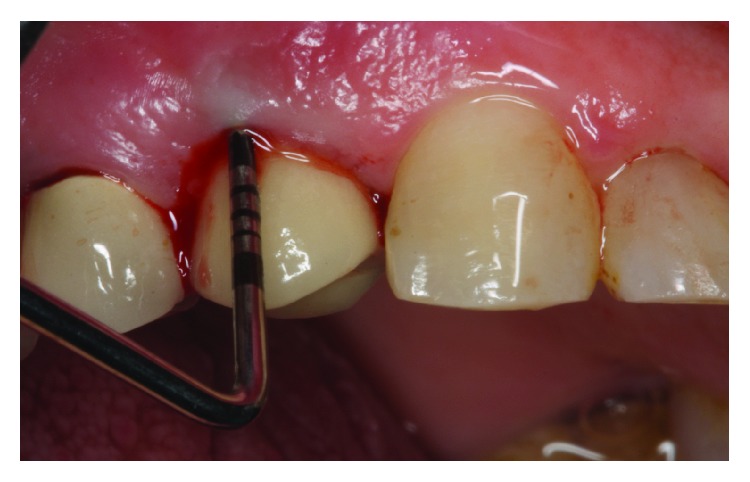
Bleeding on probing.

Intraoral observation revealed deep pockets up to 8 mm; also, profuse bleeding on probing was recorded (Figure 2). However, general periodontal condition around other teeth was good; BOP and PI around other teeth were ≤15%.

**Figure 3:**
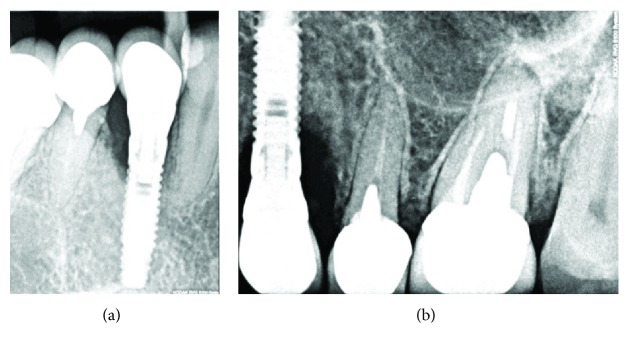
(a, b) Radiographic examination with different positions of the holder.

Radiographic examination showed severe crestal bone loss till the third thread of the implant. The bone loss pattern was characteristic to peri-implantitis. No cement remnants could be noticed; also, the bone level of adjacent teeth indicated that the patient is not periodontally involved (Figures 3(a) and 3(b)). Clinical and radiological evaluation helped to determine the diagnosis of chronic peri-implantitis.

**Figure 4:**
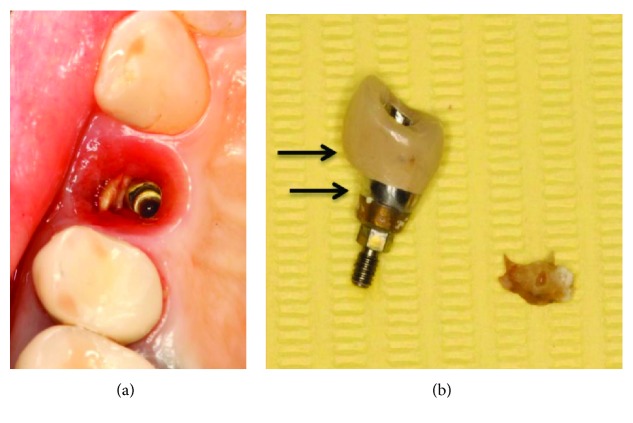
(a) Undetected cement remnants in peri-implant sulcus; (b) removed cement excess and size of undercut of the restoration.

Initial treatment plan included removal of the restoration and evaluation of the peri-implant tissues. The restoration occlusal surface was swirled through to get the access to the abutment screw. The screw was loosened, and restoration was removed. Surprisingly, a big piece of cement rest was located in the buccal site of the implant, thus invisible to radiographic examination (Figures 4(a) and 4(b)).

An extensive undercut between the cementation line on the standard abutment (lower arrow) and emergence profile of the restoration in the buccal side (upper arrow) was present (Figure 4(b)). Exactly on this side cement remnant was located in peri-implant mucosa. Thus, undercut can be described as the distance between the cementation (cement extrusion) line and the emergence profile of the restoration. It was shown that a bigger undercut results in worse cement remnant removal (Vindasiute et al. 2013).

**Figure 5:**
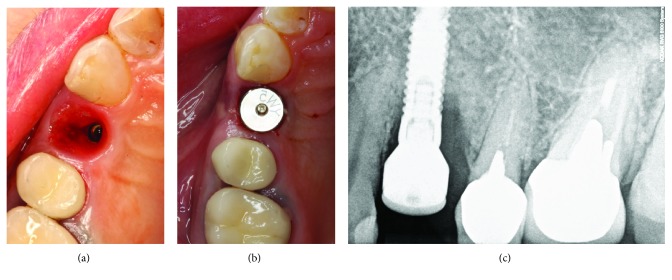
(a) Peri-implant sulcus after removal of cement excess, (b) healing abutment connection, and (c) radiographic verification.

The cement rest was removed, and peri-implant tissues were abundantly rinsed with chlorhexidine-digluconate 0.12% solution (PerioAid, Dentaid, Barcelona, Spain). There was a bedsore in peri-implant tissues, where cement remnants were resting (Figure 5(a)). Then, chlorhexidine gel (PerioAid Gel, Dentaid, Barcelona, Spain) was applied on the healing abutment, and it was tightened to the implant (Figures 5(b) and 5(c)). The excess of the gel was washed away. The patient was instructed to rinse the infected site with the same chlorhexidine-digluconate solution twice a day for 1 week.

**Figure 6:**
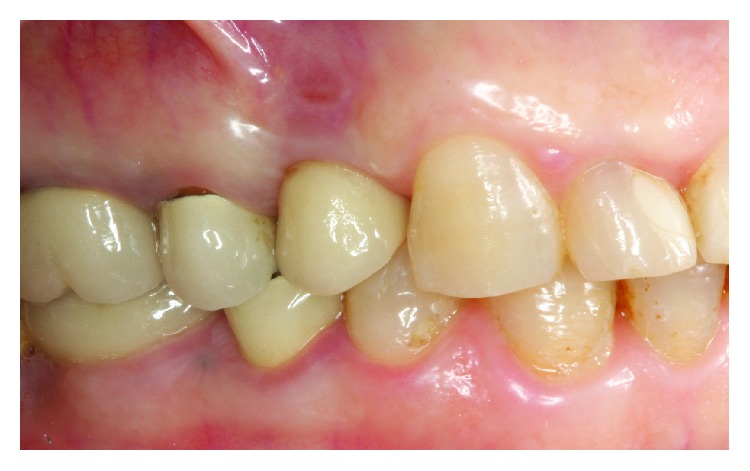
Sinus tract has healed.

The patient presented at 1 week after removal of the cement excess. The fistula was not present; the patient did not report any tenderness of the site. Clinically, soft tissues appeared healthy; there was restricted blushing of the gingiva in the place of previous fistula. The removed restoration was screwed back to the implant, and occlusal entrance was isolated with polytetrafluorethylene tape and adhesively covered with composite (Figure 6). The decision was made not to proceed with any kind of antibacterial, surgical, or regenerative treatment of peri-implantitis, and the patient was scheduled for observation visits every 6 months.

## 3. Follow-Up

**Figure 7:**
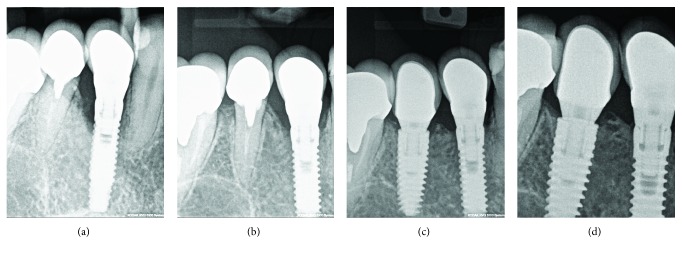
(a) Reference radiograph in 2009; (b) partial remineralization of the bone defect in 2010; (c) full reestablishment of the bone level in 2012; (d) stable bone after 10 years.

The patient presented after 1 year post removal of the cement excess without any complains. Radiological examination showed bone remineralization of the previously infected implant (Figure 7(b)). After 2 years, a full regrowth of the crestal bone around the implant was observed (Figure 7(c)). During this time, the patient had an adjacent tooth replaced with an implant. In 2019, 10 years after, a new periapical X-ray reveals stable bone around implant #24 (Figure 7(d)).

**Figure 8:**
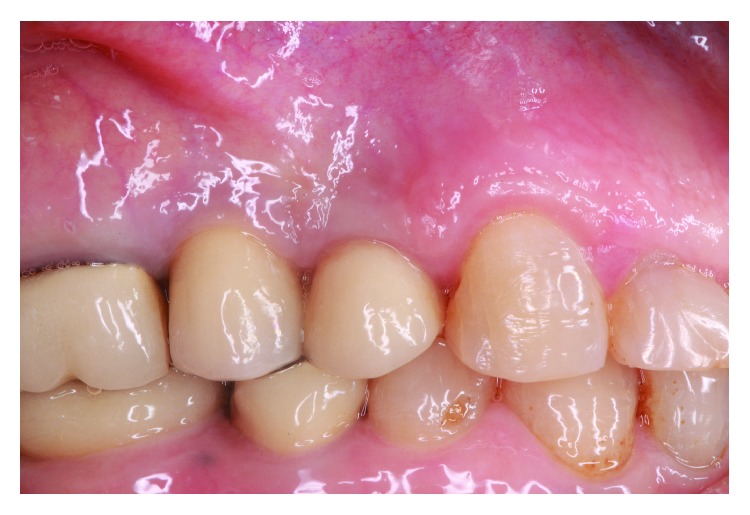
Clinical appearance of implant-supported restoration 10 years after treatment.

Clinically soft tissues were healthy; the bone contour was improved, compared to the previous situation, and only a minor change in soft tissue color remained (Figure 8).

## 4. Discussion

There was a visible crestal bone resorption due to cement remnants in this clinical case. It was shown that an individual abutment does not guarantee total cement excess cleaning at the subgingival margin [6, 7].

However, after the elimination of the residue, not only the soft tissue inflammation resolved but also bone remineralization occurred. This might be compared to remineralization of the alveolar bone around the tooth, which also can be expected. It was shown that bone regeneration occurs in infrabony pockets in patients maintained on an optimal standard of oral hygiene [8]. When infection and irritants are removed, remineralization of the bone organic matrix occurs. It can be speculated that a similar situation occurred in this implant case. Cement excess was acting like “artificial calculus,” which predisposed peri-implant disease. This case report could suggest that surgical intervention is not always necessary as a part of cement-related peri-implantitis treatment. When cement rest is removed and the patient is not periodontally compromised, remineralization of the crestal bone can occur.

The bone loss around a dental implant due to cement remnants is not direct bone loss but rather demineralization. The question arises: how long should it take to destroy all organic matrix and when to predict bone remineralization around dental implant? Perhaps, there is strong probability for a direct correlation between host response and microbiota of peri-implant sulcus.

The lack of information about bone demineralization and remineralization kinetics is also observed. It can only be speculated as a process similar to periodontal tissue changes.

Demineralization is the process caused by inflammation when mineral ions of hydroxyapatite (HA) are removed from the hard tissues, particularly in the bone [9]. Even though HA is one of the most stable calcium phosphate salts [10], the inflammation process might lead to the bone matrix changes following bone loss. Inflammation is related to the overproduction of various cytokines and bone cells [11]. It initiates hyperactivation of osteoclasts and leads to the bone degradation; also, some cytokines negatively affect osteoblast function [12]. Remineralization might be achieved by increasing osteoblast function; particularly, these cells promote crystal formation of hydroxyapatite, propagate growth in the interior part of membrane-limited matrix vesicles [13], and induce crystals in the collagenous extracellular matrix thus mineralizing bone matrix overall [14].

Recent publications regarding peri-implantitis treatment have various treatment methods in order to obtain bone remineralization, but most of them contain surgical interventions [15–19]. A study performed by Froum et al. proposed a regenerative approach for peri-implantitis treatment [16] with a consecutive series of 170 implants in 100 patients up to a 10-year follow-up. The treatment consisted of flap elevation, surface decontamination, use of enamel matrix derivative (EMD) or platelet-derived growth factor (PDGF), and guided bone regeneration with mineralized freeze-dried bone and/or organic bovine bone (with PDGF or EMD) and covered with an absorbable membrane or subepithelial connective tissue graft. In the results, it was stated that probing depth reduction averaged 5,1 mm and bone level gain averaged 1,77 mm.

While taking a look in scientific articles reporting nonsurgical treatment for peri-implantitis, the mean differences were evaluated between either presence or absence of bleeding and changes in probing depth. From the existing literature on nonsurgical therapy of peri-implantitis, it seems that limited clinical improvements have been found following mechanical therapy with ultrasonic devices or carbon-fiber curettes. In the controlled study by Karring et al. [20], peri-implantitis treatment was performed with either carbon-fiber tip with aerosol spray with hydroxylapatite or carbon-fiber curette. None of these methods in the randomized split-mouth study resulted in healing of the peri-implantitis lesions. In a randomized controlled trial comparing the use of either titanium curettes or ultrasonic device for implants, a significant reduction in bleeding and plaque scores was noted after six months, but probing depths were not improved [21]. In the clinical study performed by Sahm et al., it was noted that air-abrasive treatment resulted in significantly higher bleeding on probing reduction compared with mechanical debridement with carbon-fiber curette [22]. In the Büchter and coworker study, it is already stated that local antimicrobial treatment gives positive results in reduction of pocket probing depth of about 1,15 mm [23]. A number of case series and clinical trials have reported similar results in terms of enhancing effectiveness of peri-implantitis treatment supplemented with local antimicrobials [24–27]. The question arises: does conservative treatment of peri-implantitis lead not only to absence of inflammation and reduction of probing depth but also to remineralization around the implant site?.

The main limitation to any human clinical study is the lack of histology needed to determine the newly formed bone of the remineralization process. There is still considerable uncertainty with regard to effectiveness of the proposed treatment; in fact, further experimental investigations are needed to estimate the accurate results.

## Additional Points

*A Key Clinical Message*. Bone remineralization around dental implants with a history of peri-implantitis is possible after irritant factors are removed and conservative treatment is performed.
